# Audience of Academic Otolaryngology on Twitter: Cross-sectional Study

**DOI:** 10.2196/25654

**Published:** 2021-12-08

**Authors:** Deborah X Xie, Emily F Boss, C Matthew Stewart

**Affiliations:** 1 Department of Otolaryngology-Head and Neck Surgery Johns Hopkins University Baltimore, MD United States

**Keywords:** Twitter, otolaryngology, residency, medical education, social media, internet

## Abstract

**Background:**

Despite the ubiquity of social media, the utilization and audience reach of this communication method by otolaryngology-head and neck surgery (OHNS) residency programs has not been investigated.

**Objective:**

The purpose of this study was to evaluate the content posted to a popular social media platform (Twitter) by OHNS residency programs.

**Methods:**

In this cross-sectional study, we identified Twitter accounts for accredited academic OHNS residency programs. Tweets published over a 6-month period (March to August 2019) were extracted. Tweets were categorized and analyzed for source (original versus retweet) and target audience (medical versus layman). A random sample of 100 tweets was used to identify patterns of content, which were then used to categorize additional tweets. We quantified the total number of likes or retweets by health care professionals.

**Results:**

Of the 121 accredited programs, 35 (28.9%) had Twitter accounts. Of the 2526 tweets in the 6-month period, 1695 (67.10%) were original-content tweets. The majority of tweets (1283/1695, 75.69%) were targeted toward health care workers, most of which did not directly contain medical information (954/1283, 74.36%). These tweets contained information about the department’s trainees and education (349/954, 36.6%), participation at conferences (263/954, 27.6%), and research publications (112/954, 11.7%). Two-thirds of all tweets did not contain medical information. Medical professionals accounted for 1249/1362 (91.70%) of retweets and 5616/6372 (88.14%) of likes on original-content tweets.

**Conclusions:**

The majority of Twitter usage by OHNS residency programs is for intra and interprofessional communication, and only a minority of tweets contain information geared toward the public. Communication and information sharing with patients is not the focus of OHNS departments on Twitter.

## Introduction

Social media continues to be a growing and evolving aspect of daily life for the general population. Over the last 15 years, the percentage of US adults who use at least one social media website has increased from 5% to 72% [1]. Online resources and social media platforms hold significant potential as methods of communication and information dissemination between health care providers and their patients.

With the development of electronic medical records, many hospital systems allow for patients to contact their providers and access records through an online patient portal [2]. Younger patients are more likely than their older counterparts to use these portals in the orthopedic [3] and cancer [4] patient populations. There is a similar correlation of social media usage with age, as a higher proportion of younger adults are using social media (90% of individuals aged 18-29 years) compared to older adults (40% of individuals over the age of 65 years) [1].

With the ever-expanding role of telemedicine in patient care, particularly during the COVID-19 pandemic, we must be mindful of opportunities for patient engagement and education outside of the office. With its rising ubiquity, the utilization and audience reach of social media by medical professionals is an emerging field of research. Twitter is a popular platform that has proven to be useful in academic networking [5-8]. In the field of otolaryngology-head and neck surgery (OHNS), Twitter has been studied as a patient resource for information about tonsillectomy [9], cochlear implantation [10], and hearing loss [11]. However, there have been no investigations into the use of this social media platform by academic OHNS residency programs. Thus, the purpose of this study was to evaluate the content and target audience of academic OHNS residency programs on Twitter.

## Methods

Data for this cross-sectional study were collected in August 2019 from Twitter (Twitter Inc, San Francisco, CA). OHNS residency programs were included if they were accredited by the Accreditation Council for Graduate Medical Education (ACGME). Twitter accounts were identified by searching each program’s website for profile links as well as by searching for the name of the program directly on Twitter. Accounts that were division-specific were excluded.

**Figure 1:**
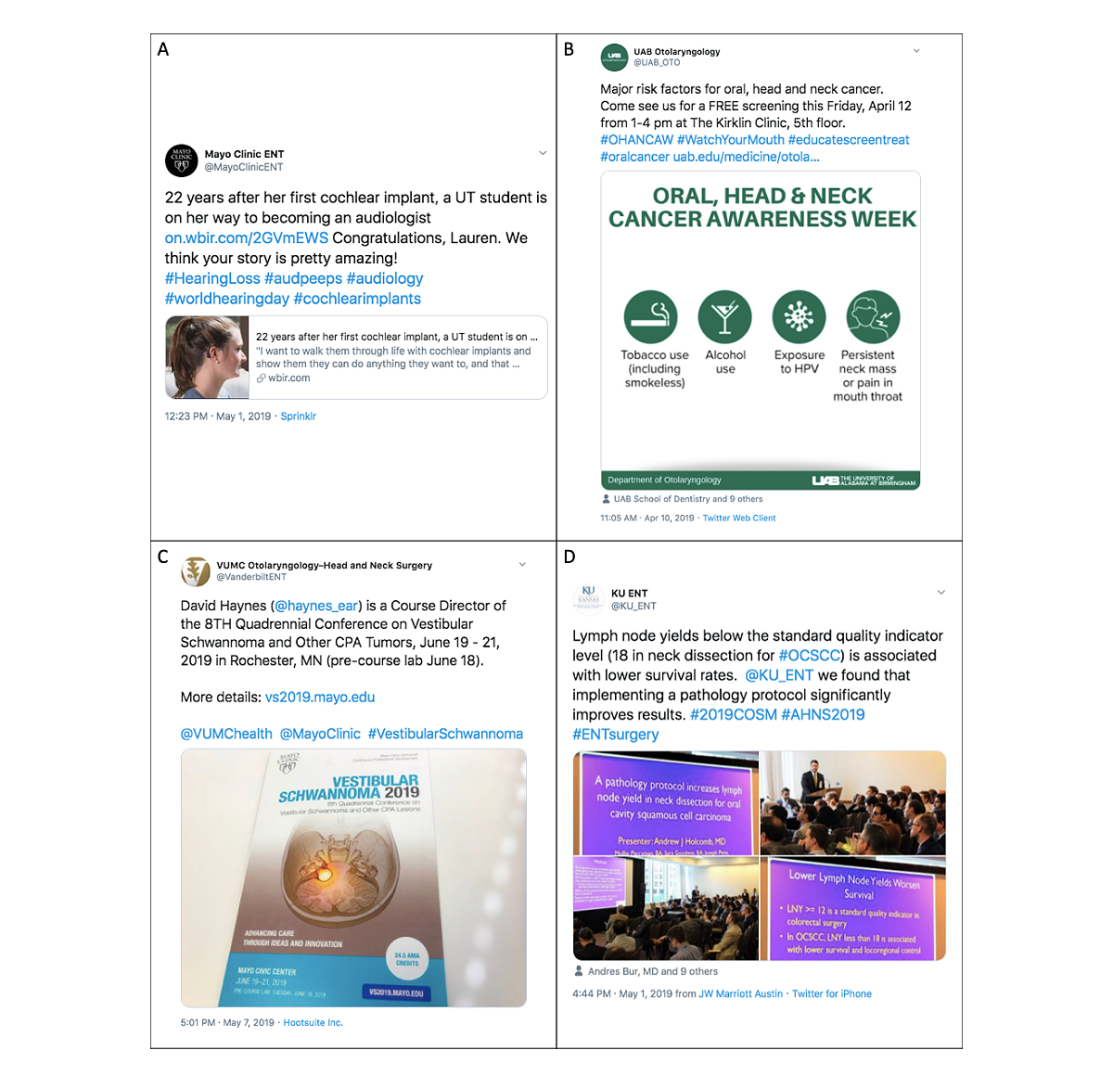
Sample tweets demonstrating original content (A) targeted toward patients and contained no medical information (ie, uninformative), (B) targeted toward patients and directly containing medical information, (C) targeted toward medical professionals and uninformative, and (D) targeted toward medical professionals and directly containing medical information.

Twitter metrics (number of tweets, number of followers, and accounts being followed by the program) and tweets from the last 6 months were downloaded with Twitonomy (Diginomy Pty Ltd, New South Wales, Australia). A content analysis of all individual tweets from these accounts during the 6-month period from March to August 2019 was also performed. The text of each tweet was categorized for origin of content (original text created by the account versus retweet of another user’s content), level of information (directly informative, indirectly informative by providing a link or web address for additional information, or uninformative), and target audience (health care worker versus general public). For example, a tweet promoting a grand rounds session would be categorized as original content, uninformative, and targeting health care workers (Figure 1).

To further characterize the information communicated in the tweets, a sample of 100 tweets was analyzed to identify common themes, which was then applied to categorize additional tweets. This sample of tweets was selected with a random number generator. The total number of likes or retweets each tweet received by health care professionals was also quantified to characterize the population of users interacting with published tweets. Users were categorized as health care professionals if their Twitter profile listed their profession or if they were listed as an employee on an institutional website. These individuals included physicians, nurses, physician assistants, nurse practitioners, speech-language pathologists, and audiologists.

Data analyses and descriptive statistics were performed using R version 3.6.2 software (Vienna, Austria). Difference in social media metrics were determined by the *χ^2^* test.

## Results

Of the 121 ACGME-accredited residency programs, 35 (28.9%) had Twitter accounts (Table 1). Twenty-six (74.3%) of these were active during the study period. A total of 2526 tweets were published during the study period. Programs published a median of 69 tweets (IQR 34-157). Over half of the tweets (1330/2526, 52.65%) from the study period were written by four accounts (Vanderbilt University, University of Kansas, University of North Carolina, University of Nebraska). Tweets were retweeted a total of 14,970 times (range 0-2603; median 1, IQR 0-2) and liked 46,988 times (range 0-9014; median 4, IQR 1-8).

**Table 1:** Twitter metrics of programs that were active during the study period.

Program	Twitter handle	Total number of tweets	Number of accounts following	Number of followers
Baylor College of Medicine	BCM_Oto	560	340	839
Cleveland Clinic	CCF_ent_program	28	12	83
Duke University	Duke_Oto	155	55	150
Columbia University	ColumbiaOto	121	611	287
Georgetown University	georgetownOTO	6	147	55
Henry Ford Hospital	henryfordent	94	116	51
Mayo Clinic (Rochester)	MayoClinicENT	169	312	168
Medical College of Wisconsin	Mcwent	155	65	491
Northwestern University	NM_ENT	273	208	140
Penn State Health	WeAreOto	346	476	910
Southern Illinois University	SIU_ENT	60	58	156
University of California, Davis	UCDAVIS_OTOHNS	211	168	412
University of Alabama	UAB_OTO	287	144	244
University of Arizona	UofAENT	246	246	471
University of Arkansas	UAMSENT	147	9	65
University of Florida	UFOtolaryngolo1	56	28	66
University of Kansas	KU_ENT	1291	805	1060
University of Michigan	UMichOto	1128	186	757
University of Minnesota	ent_umn	88	75	326
University of Missouri	MizzouENT	135	20	147
University of Nebraska	EntUnmc	281	514	160
University of North Carolina	unc_ent	484	595	995
University of Virginia	uvaotohns	1356	23	712
Vanderbilt University	vanderbiltENT	1990	1697	2099
Washington University in St. Louis	WUSTL_ENT	111	125	158
Yale	Yale_ENT	101	221	166

Residency program accounts published 1695/2526 (67.10%) tweets of original content, and the remaining 32.90% (831/2526) of tweets were retweets or republication of another user’s content. Original-content tweets were subsequently retweeted by other Twitter users 1362 times (range 0-15; median 0, IQR 0-1) and liked 6372 times (range 0-48; median 2, IQR 1-5). Medical professionals accounted for 1249/1362 (91.70%) of retweets and 5616/6372 (88.14%) of likes on original tweets. The majority of tweets (1283/1695, 75.69%) contained information targeted for health care workers, and included tweets describing recent publications, grand rounds, and new hires. The remaining 24.31% (412/1695) of tweets were targeted toward patients or the general public, and included tweets on recommended cancer screening protocols, patient testimonials, news stories, and cancer awareness months.

The majority of original tweets were uninformative and did not contain any medical information (1130/1695, 66.67%). Only 116 of original tweets (6.84%) directly contained medical information and an additional 449 tweets (26.49%) indirectly provided medical information by including links to external websites with medical information. Tweets targeted toward the general public were more likely to directly contain medical information (16.5% vs 3.7%, *P*<.001; relative risk [RR] 4.41, 95% CI 3.1-6.28). Conversely, tweets targeted toward physicians were more likely to be uninformative (74.4% vs 42.7%, *P*<.001; RR 1.74, 95% CI 1.55-1.96).

A random sample of 100 posts were analyzed to identify content themes (Table 2). Given that the largest sample of tweets (n=954) were targeted toward medical professionals and uninformative, these tweets were then coded into the identified themes. Trainees and education were the most common subject of these tweets, followed by participation at conferences and research publications.

**Table 2:** Content themes identified among tweets targeting medical professionals that were uninformative (n=954).

Tweet content category	Description	Tweets, n (%)	Representative post
Awards and grants	Tweets featuring recipients of grant funding or awards	56 (5.9)	Congratulations to @MichaelPitmanMD and his team for being awarded a $3M #R01 #grant by the @NIH for their research on vocal fold paralysis, “Mechanisms of axon guidance in laryngeal reinnervation following injury of the recurrent laryngeal nerve.” Amazing!!! #laryngology #voice [@ColumbiaOto]
Conference attendance or presentation	Tweets sharing poster/oral presentations, panelists, or attendance at academic conferences	263 (27.6)	Dr Kathleen Yaremchuk is in Germany! She's presenting on Sleep Apnea at the 90th annual Germany meeting for Otolaryngologists. #medtwitter #Doctors #WomeninMedicine [@henryfordent]
Grand rounds and lectures	Tweets highlighting topics of grand rounds or lectures	85 (8.9)	Join us tomorrow at 7AM for our ENT Grand Rounds. Taylor Riall, MD will be presenting a talk entitled “Maintaining the Fire: Wellbeing, Resilience & Intentional Culture. Livestream here: https://t.co/6seb90cH82 #uofaent #otolaryngology [@UofAENT]
Networking and promotion	Tweets promoting the connection of individuals or departmental events	89 (9.3)	Lots of awesomeness @KU_ENT Here's a few more who are on Twitter: @Mollie_Perryman @amyjacks13 @jplepse @smchale3 @wichova_md @AndrewJHolcomb @MattyShews @syalamanchaliMD [@KU_ENT]
Research and publications	Tweets sharing research projects and publications	112 (11.7)	Dr Paul Russell has a new paper with two of @VanderbiltU's Mechanical Engineering researchers: ”A multi-subject accuracy study on granular jamming for non-invasive attachment of fiducial markers to patients. [@VanderbiltENT]
Training and education	Tweets focusing on medical students, residents, fellows, and educational efforts	349 (36.6)	Resident training lights up our surgical simulation lab #temporalbonelab #ENT #otolaryngology #stateoftheart @ear_wick [@WUSTL_ENT]

## Discussion

### Principal Findings

In this study, we reviewed and analyzed the usage patterns of academic OHNS residency programs on Twitter. Thirty-five programs had accounts on Twitter at the time of this analysis, which represents more than double the 14 programs that were on Twitter in April 2017 [12]. Interestingly, 4 programs (11% of the programs on Twitter) were responsible for over half of the tweets produced in our 6-month study period. A recent investigation by the Pew Research Center found that the most active 10% of Twitter users produce 80% of all tweets [13]. These data are likely skewed by the number of inactive users or “bot” accounts (automated accounts that post content based on algorithms, as opposed to a human-run account). Although moderately imbalanced, the activity of the OHNS community is more equitable compared with the activity of the entire Twitter population. Approximately 25% of programs with Twitter accounts did not publish any tweets during the study period. It is possible that the individuals responsible for managing these accounts are no longer employed by the institutions, or perhaps the accounts have been neglected since their creation.

The current use of Twitter in the academic OHNS community is focused on intra and interprofessional communication. The content included in these tweets reflects topics of trainees and education, presentations at academic conferences, and research publications. These findings are consistent with previously published studies in other fields of medicine [6,7,14,15]. Medical professionals provided the majority of interactions with tweets by OHNS residency programs, accounting for 97.1% of retweets and 88.1% of likes. Even though approximately one-quarter of the tweets analyzed in this study were targeted toward patients and the general public, the overwhelming majority of interactions with the tweets were provided by health care professionals, suggesting that the general public is not interacting with the content that is curated for them. Additionally, very few of these tweets directly contained medical information that provides patient education. In a 2017 study, 43% of tweets by urology departments were directed at physicians [16], which was lower than the rate observed in this study for the OHNS community. This relationship may vary in each field of medicine, as Kloth et al [17] observed fewer interactions between pain patients and their providers on Twitter compared to oncology patients. These findings confirm that Twitter is not the currently preferred medium of communication for information dissemination to patients. The reason behind these patterns is unknown, although possible factors include patients preferring other online/social media platforms as medical resources, fear of misinformation, or personal privacy concerns. Future studies may focus on understanding patient preferences for the communication of medical information on social media.

Although Twitter does not seem to be a favorable network for patient communication, it efficiently serves as a professional networking medium. Twitter has been used to supplement academic conferences and disseminate information to a broader audience [18-20]. Moreover, maintaining an active social media presence to promote department activity may improve a department’s reputation. Both US News and World Report and Doximity ranking systems include program reputation [21,22], and have previously been associated with program social media presence in OHNS and other fields [12,20,23]. In a multi-institutional survey of surgeons, 70% indicated they believe that social media benefits professional development [24]. This may be of particular importance for women and underrepresented minorities in medicine who face unique challenges in their academic careers, as Twitter provides a network of mentors and peers who may otherwise be inaccessible [25,26]. Moreover, these networks may be utilized by residency applicants to garner information about prospective programs, particularly as the COVID-19 pandemic has affected the residency application process [27-29]. Given the lack of away rotations or in-person interviews, students may be spending more time on social media searching for information compared to previous years. In a survey-based study, Oyewumi et al [30] reported that almost 60% of Canadian otolaryngologists utilize social media but most were unsure how to apply these tools to their practice. As our understanding of social media in medicine continues to develop, hospitals and OHNS departments may consider incorporating social media training into their educational curriculum to ensure that their health care providers are optimizing the use of these platforms.

Beyond Twitter, new social media platforms are constantly being developed and popularized, providing new methods to disseminate health information. For example, TikTok is an app that allows users to upload video clips up to 60 seconds long with music, text, and filters. A few physicians have turned to this platform, particularly targeting teenage populations, to provide health education and combat misinformation on topics such as birth control, vaping, and vaccination [31,32]. Additionally, there are patient-specific online networking sites such as PatientsLikeMe, which specifically attract patients with a common condition to connect with other individuals and gather information about their disease, available treatments, and treatment side effects [33,34]. Facebook groups have been shown to be useful platforms for patients with idiopathic subglottic stenosis to share resources, personal experiences, and emotional support [35]. These platforms highlight areas of information need, and may improve communication and information dissemination from health care providers. Social media platforms also hold promise to recruit patients for research endeavors [36].

### Limitations

There are a few limitations to this study. Many individual otolaryngologists are active on Twitter; however, these accounts were not included in this analysis, as we focused on the activity of residency programs over individuals. Furthermore, private practice groups and academic institutions without residency programs were not included, and the content of their social media presence was not captured. To facilitate recruitment of medical students during the COVID-19 pandemic, some institutions have created separate, resident-led social media accounts distinct from preexisting departmental accounts, and these two groups have overlapping but separate target audiences. Patients may not be interested in the hobbies and social events of residents, whereas this is essential information for medical students. Conversely, departments may be able to advertise with testimonials or education materials to attract new patients. The data in this study were collected prior to the pandemic and, to our knowledge, no institutions had multiple Twitter accounts at the time of data analysis. However, future studies may consider how these groups utilize different social media platforms to effectively reach their target audience. When coding tweets based on theme, some tweets contained information that included more than one theme. For example, a tweet describing a resident’s presentation at a conference describes both a trainee and conference participation. Each tweet was ultimately coded to only one theme based on the primary message conveyed in the tweet, and this must be taken into account when interpreting the data. Finally, given the cross-sectional nature of this analysis, we were not able to assess any temporal changes in social media presence.

### Conclusion

Social media is ubiquitous and presents a unique communication medium within the health care industry. The majority of Twitter usage by OHNS residency programs is for intra and interprofessional communication. Only a minority of tweets contain information geared toward the general public, highlighting that communication and information sharing with patients is not the current focus of OHNS residency programs on Twitter.

## Abbreviations

ACGME: Accreditation Council for Graduate Medical Education
OHNS: otolaryngology-head and neck surgery
RR: relative risk
